# Automation of absolute protein-ligand binding free energy calculations for docking refinement and compound evaluation

**DOI:** 10.1038/s41598-020-80769-1

**Published:** 2021-01-13

**Authors:** Germano Heinzelmann, Michael K. Gilson

**Affiliations:** 1 Departamento de Física, Universidade Federal de Santa Catarina, Florianópolis, Santa Catarina Brazil; 2 Skaggs School of Pharmacy and Pharmaceutical Sciences, University of California, San Diego, USA

**Keywords:** Biochemistry, Computational biology and bioinformatics, Drug discovery

## Abstract

Absolute binding free energy calculations with explicit solvent molecular simulations can provide estimates of protein-ligand affinities, and thus reduce the time and costs needed to find new drug candidates. However, these calculations can be complex to implement and perform. Here, we introduce the software BAT.py, a Python tool that invokes the AMBER simulation package to automate the calculation of binding free energies for a protein with a series of ligands. The software supports the attach-pull-release (APR) and double decoupling (DD) binding free energy methods, as well as the simultaneous decoupling-recoupling (SDR) method, a variant of double decoupling that avoids numerical artifacts associated with charged ligands. We report encouraging initial test applications of this software both to re-rank docked poses and to estimate overall binding free energies. We also show that it is practical to carry out these calculations cheaply by using graphical processing units in common machines that can be built for this purpose. The combination of automation and low cost positions this procedure to be applied in a relatively high-throughput mode and thus stands to enable new applications in early-stage drug discovery.

## Introduction

Protein-ligand binding free energy calculations based on atomistic molecular simulations promise to play a growing role in drug discovery, as they provide estimates of the binding affinities of compounds proposed as drug candidates for a protein target, and thus may reduce the time and cost required for trial-and-error experimentation^1,2^. Thus, in settings where the calculations are sufficiently fast and accurate^3,4^, one may anticipate significant savings of time and cost in early stages of drug discovery^5–8^. It is informative to divide this broad class of methods into two subtypes: relative binding free energy (RBFE) calculations^9–14^; and absolute binding free energy (ABFE) calculations^15–25^.The former, RBFE, estimates the difference in binding free energy between two compounds by computing the change in free energy associated with a non-physical transformation of one compound to the other, in the binding site and in bulk solvent^9,26^. Since it is easiest to carry out such alchemical transformations between compounds that are similar to each other and that adopt similar bound poses, RBFE calculations are often regarded as particularly suitable for the lead-optimization stage of drug discovery, where small chemical modifications of the initial lead compound must be selected. Interestingly, though, a recent study reports greater impact on the earlier hit-to-lead stage^4^.

In contrast, ABFE calculations estimate the standard free energy of binding of a single compound to a protein by considering a process in which the compound is removed from the binding site into bulk solvent. This can be accomplished by a non-physical (i.e. alchemical) decoupling pathway^15,16,27^, in which the ligand is, in effect, decoupled from the binding site and recoupled with bulk solvent; or by modeling the physical process of moving the fully coupled ligand out of the binding site^18,23,24,28^ into solvent. It is worth noting that any valid ABFE method must account for the free energy of releasing the ligand to bulk solvent at standard concentration^16^.

Because ABFE calculations do not require the alchemical conversion of one compound into another similar compound, they are better suited to the task of screening diverse compounds for the ability to bind a targeted protein^13,20,24,27,29^. However, using ABFE methods to screen compounds is complicated by the fact that the free energy simulations need to sample the correct ligand pose in order to give a correct result. This is problematic, because current docking methods cannot reliably provide the correct pose as a starting point for the simulations, and a typical MD simulation of a bound ligand cannot move the ligand from an incorrect initial pose to the correct one, due to the high energy barrier blocking any large conformational change. Encouragingly, several prior studies have shown that ABFE calculations may be used to determine the relative stability of various plausible binding modes of a given ligand^19,20,24^. A second obstacle to the use of ABFE methods is that the standard double-decoupling (DD) method^16^ leads to changes in the net charge of the simulated system when the ligand has a nonzero net charge, and this can lead to numerical artifacts which may be difficult and/or time-consuming to correct^30–32^.However, this problem does not arise with the attach-pull-release (APR) method^23,24^, because the ligand remains within the simulation box, and, as discussed below, the DD method may be modified to have the same advantage. Thus, we have the possibility of a workflow in which ABFE calculations are carried out for multiple plausible poses provided by a docking program, and the results are synthesized to provide information on which poses are most stable and on the overall binding free energy of the ligand. The present study is a step toward this goal.

The use of ABFE calculations to enhance pose-prediction and virtual screening has become increasingly attractive with recent increases in the time scales accessible by MD simulations, particularly through the use of inexpensive Graphics Processing Units (GPUs)^33–36^. However, the widespread adoption of these methods has remained limited by the substantial amount of human effort needed to use them. Key steps include assignment of force field parameters, construction of initial system configurations, setup of conformational restraints, equilibration of the simulation windows, execution of the production runs, and analysis of the results. Important prior contributions toward automation of ABFE calculations include the CHARMM-GUI server^37,38^ and the binding free energy estimator (BFEE)^39,40^. The former is a web-based interface that helps create input files for the various stages listed above. The latter is a tcl plug-in for VMD^41^, with a graphical interface that creates a complete ABFE workflow starting from an initial prepared and equilibrated protein-ligand complex. However, these tools do not automate virtual compound screening with ABFE calculations. In addition, there would be great value in an open-source package written in the flexible and widely used Python programming language, as this would facilitate use, replication, customization and extension of the method.

Here, we aim to prove the principle that ABFE calculations for diverse ligands can be effectively automated. To this end, we report Binding Affinity Tool (BAT), an open-source Python package to facilitate and automate the use of ABFE calculations for virtual compound screening. The BAT package enables automated computation of the binding free energy of a series of diverse ligands to a chosen receptor with minimal manual intervention, starting only from the coordinates of one or more co-crystal structures or docked complexes. It can be used for both pose refinement and ligand ranking. To maximize computational throughput, BAT takes advantage of the high computational performance of AMBER’s *pmemd.cuda* sofware^42,43^ on GPUs. The package is designed for use in the early stages of drug discovery, with the aim of using computation to reduce the time and cost of experimentation. The BAT package supports three ABFE methods:

- The double decoupling (DD) method^16^ involves computing the work of decoupling the ligand from the binding site and the work of decoupling the ligand from pure solvent. It is well suited to charge-neutral ligands in both surface and deeper binding sites. However, for ligands with nonzero net charge, the decoupling processes cause changes in the net charge of the entire simulation system. These can lead, in turn, to undesirable numerical artifacts whose correction requires additional calculations^30,31,44^.
- The attach-pull-release (APR) method^23,24^ computes the work of unbinding along a physical pathway in which the ligand is removed stepwise from the binding site. This method does not change the net charge of the system and hence avoids the numerical artifacts mentioned above. However, it is harder to use for ligands in non-surface binding sites because, in that case, constructing a physical pathway to the solvent can cause substantial perturbations of the protein’s structure.
- The simultaneous decoupling and recoupling (SDR) method^27^ uses nonphysical, alchemical pathways to extract the ligand from the binding site. Unlike DD, however, SDR recouples the ligand with bulk solvent at a distance from the protein at the same time as it decouples the ligand from the binding site, so the net charge of the system remains constant during the transfer process. Thus, the SDR method combines the advantages of both methods described above, being suitable to neutral or charged ligands, with or without clear access to the solvent.

This paper briefly reviews the theory of ABFE calculations, explains how this theory is implemented in the BAT package, tests the applicability of BAT to several protein-ligand systems, characterizes performance, and discusses future applications.

**Figure 1:**
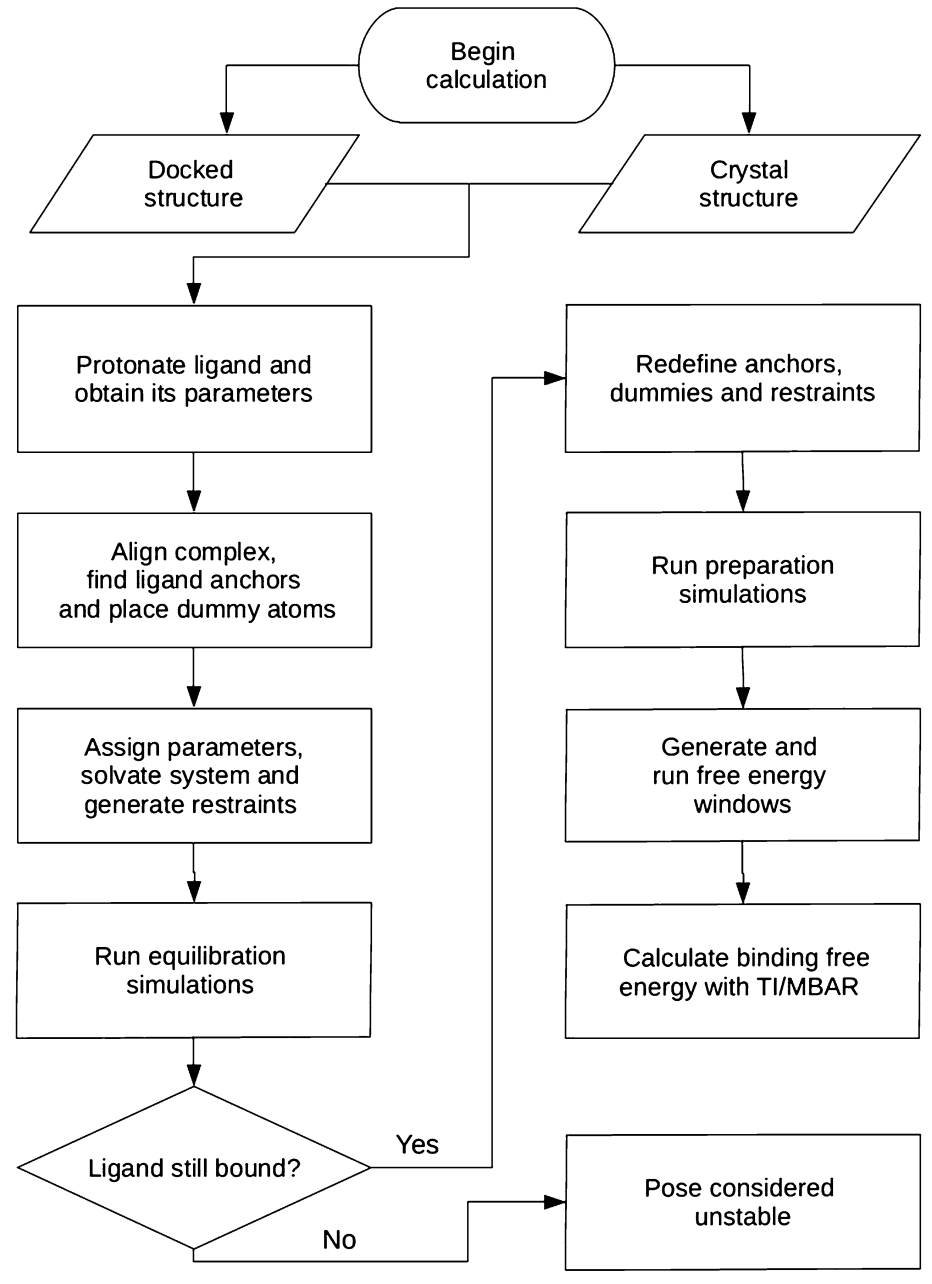
Workflow of the BAT.py software. See text for details.

## Functionality and workflow of BAT

The primary input to BAT is one or more three-dimensional structures of a given ligand and protein, each with a different pose of a given ligand and, potentially, a distinct binding-site conformation. These structures may be experimentally determined cocrystal structures and/or crystal structures of the protein with ligand poses generated by a docking algorithm. The software also requires files specifying the force field parameters to use in the simulations, and a BAT input file containing parameters such as those defining the restraints (below), the dimensions of the simulation box, and the MD timestep. A single BAT input file can be used for multiple protein-ligand input structures, including varied poses and ligands, without additional user intervention. The output of the BAT run for a given ligand and protein is a file containing the predicted binding free energy of the ligand for each input pose. The pose with the most favorable binding free energy then is predicted as the most stable pose, and the overall binding free energy can be computed from the free energies $\Delta G^\circ _i$ of the $N_{pose}$ individual poses *i*:

1 $$\begin{aligned} \Delta G^\circ _{bind} = -RT \ln \sum _i^{N_{pose}} e^{-\beta \Delta G^\circ _{i}} \end{aligned}$$

where *R* is the gas constant, *T* is absolute temperature, and $\beta ^{-1}=RT$. The BAT package is written in Python and uses a number of other software tools. These include OpenBabel^45^ for ligand protonation, VMD^41^ to help assemble and orient the proten-ligand complexes, MUSTANG^46^ for protein alignment, AMBERTools^43^ to assign force field parameters and build simulation systems, and AMBER’s *pmemd.cuda*^34^ to run the MD simulations. The BAT.py software is is available https://github.com/GHeinzelmann/BAT.py.

The overall flow of the algorithm (Fig. 1) starts with the input structures. The first step is to assign protonation states and force field parameters to the ligand. Next, the input protein structure is aligned with a reference structure of the same protein, or a similar one, so it has the correct orientation for the various restraints to be applied. These restraints require anchor atoms on the protein and ligand, as well as three dummy atoms, which are positioned based on the input structure. Protonation states and force field parameters are then assigned to the protein, and the system is solvated with water molecules, ions needed for electroneutrality and, optionally, additional ions to set a desired ionic strength. Protein and ligand restraints, detailed below, are defined and imposed, and an initial equilibration simulation is carried out for each pose, while the ligand restraints are gradually released to generate starting configurations for the subsequent free energy calculations. If the ligand starting from a given pose leaves the binding site at this stage, the pose is deemed unstable, and no free energy calculation is done for it. This filter saves time by terminating calculations for ligands or poses that are clearly unstable. Additional simulations are then run to prepare the windows needed for the binding free energy calculations for each pose. The free energy calculations are then executed for all poses and the results are saved to the output file. If the input has multiple ligand poses, all are processed in the same manner.

## Theoretical framework

This section provides the theoretical rationale for the computational procedures. For simplicity, we consider here the binding free energy of a single pose. When multiple poses are considered for a ligand, these may be combined to the overall free energy according to Eq. (1).

The dissociation constant ($K_d$) of a ligand-protein complex, LP, to free ligand, L, and protein, P, is related to the binding free energy by the expression^16^:

2 $$\begin{aligned} K_d = \frac{[L][P]}{[LP]C^\circ } = e^{\Delta G^\circ _{bind}/R T} , \end{aligned}$$

where R is the gas constant, $C^\circ$ is the standard concentration of 1 M, [L], [P] and [LP] are the equilibrium concentrations of the respective species, and ${\Delta G^\circ _{bind}}$ the standard binding free energy of the two molecules. In principle, the quantities [L], [P] and [LP] could be obtained from time- or ensemble-averaged ergodic simulations sampling bound and unbound states, enabling direct evaluation of $K_d$ and hence ${\Delta G^\circ _{bind}}$. In practice, this direct approach is usually computationally intractable because of the low rate constants for the binding and unbinding processes^47^.

**Figure 2:**
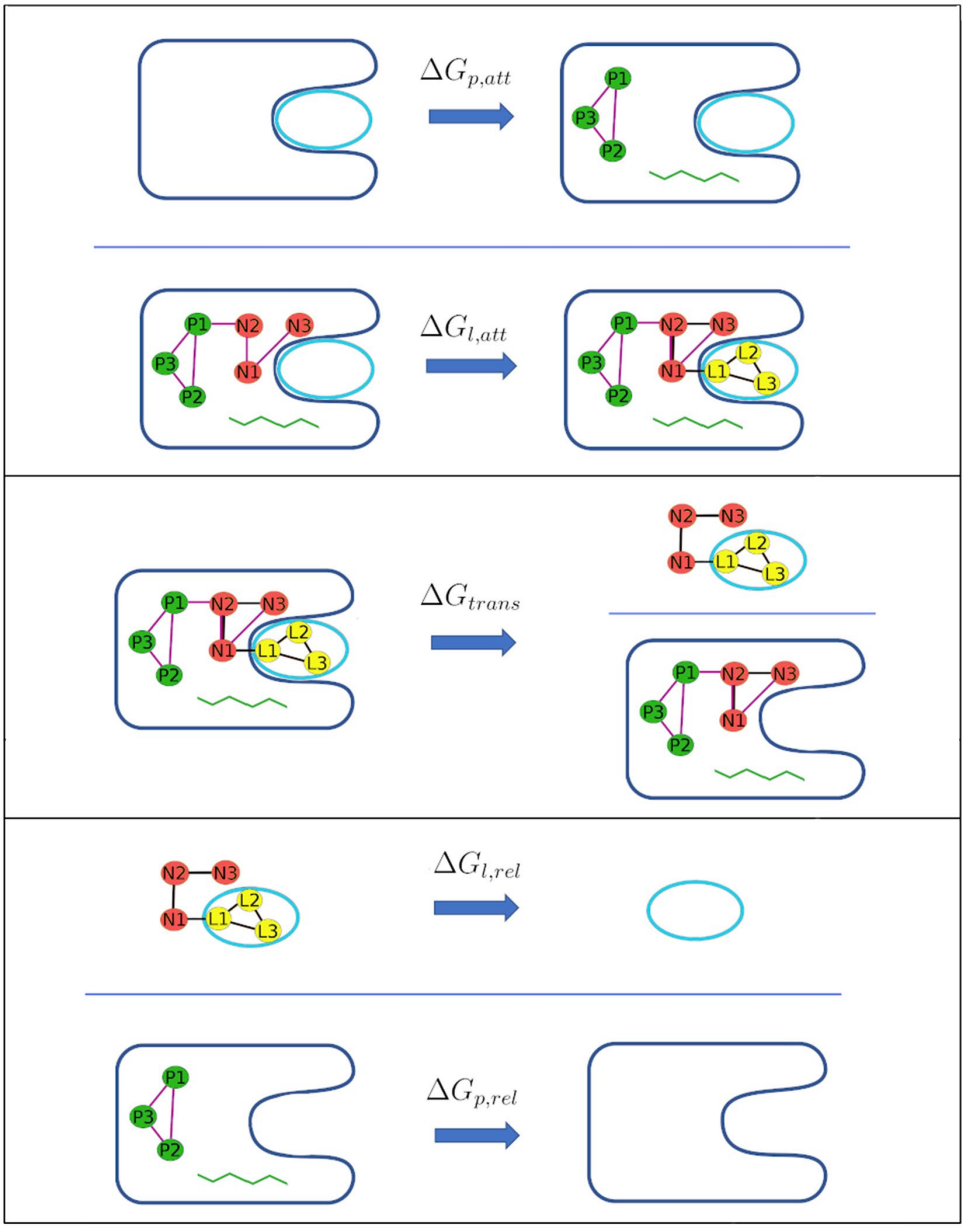
(Top panel) Attachment of restraints, first to the protein and then the ligand. The protein conformational restraints are denoted by the green squiggle. (Middle panel) Transfer ligand from binding site to bulk solvent, using the double decoupling method. (Bottom panel) Release of the ligand and protein restraints in the unbound state. P1, P2 and P3 indicate protein anchor atoms; N1, N2 and N3 indicate artificial dummy atoms whose locations are fixed in the lab frame; and L1, L2 and L3 indicate ligand anchor atoms.

To overcome this limitation, one may instead obtain ${\Delta G^\circ _{bind}}$ in terms of the reversible work of a dissociation process forced by artificial restraints. This dissociation process connects the bound and dissociated states with intermediate states along a pathway that may be either non-physical (“alchemical”) or physical. Either way, the overall free energy of binding may be written as a sum of terms, each corresponding to a step illustrated in Fig. 2:

3 $$\begin{aligned} -\Delta G^\circ _{bind} = \Delta G_{p,att} + \Delta G_{l,att} + \Delta G_{trans} + \Delta G_{l,rel} + \Delta G_{p,rel} \end{aligned}$$

Above $\Delta G_{p,att}$ and $\Delta G_{l,att}$ represent the reversible work of attaching restraints, first to the protein, and then to the ligand in the context of the resulting restrained protein; $\Delta G_{trans}$ is the reversible work of transferring the restrained ligand from the restrained protein into bulk solvent; and $\Delta G_{p,rel}$ and $\Delta G_{l,rel}$ are the reversible work of then releasing the restraints that were attached in the initial steps. Note that, because the ligand and protein have negligible interactions with each other following the transfer step, the values of the two release free energies are independent of each other. The BAT software supports two alchemical methods and one non-alchemical method of computing the transfer free energy, ${\Delta G_{trans}}$.

**Figure 3:**
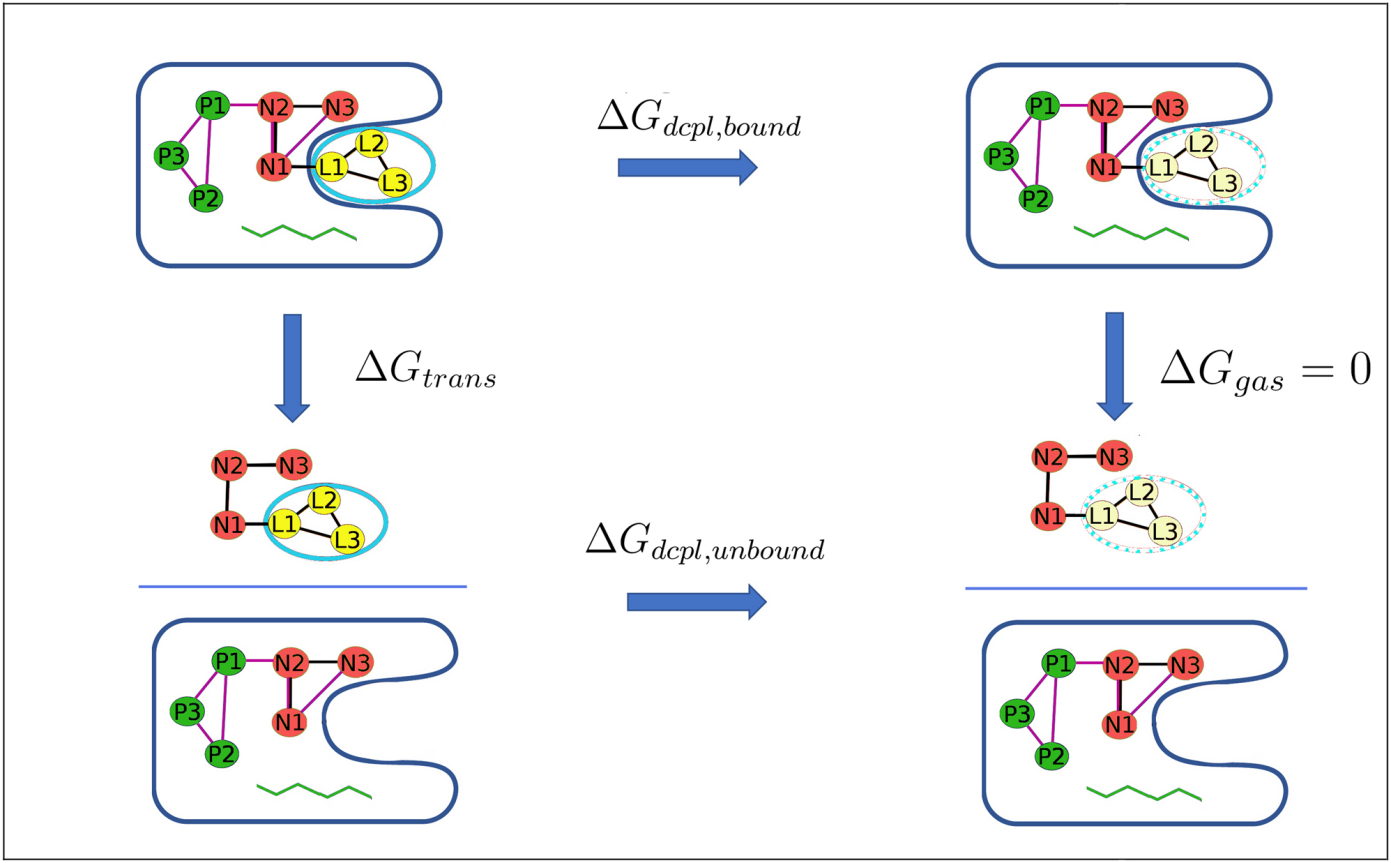
Thermodynamic cycle showing the calculation of $\Delta G_{trans}$ using the DD method, in which the restrained ligand is brought to the gas phase, both in the bound and unbound states.

The first alchemical method is the original double decoupling (DD) method^16^, which involves computing the reversible work, and hence the free energy change, of two processes. In the first, the bound ligand is gradually decoupled from the protein and solvent to yield a ligand in the gas phase, with work $\Delta G_{dcpl,bound}$. In the second, the free ligand is gradually decoupled from just the solvent to yield, again, a ligand in the gas phase, with work $\Delta G_{dcpl,unbound}$. The state of the final gas-phase ligand is the same in both decoupling calculations, so the transfer term may be computed as (Fig. 3):

4 $$\begin{aligned} \Delta G_{trans} = \Delta G_{dcpl,bound} - \Delta G_{dcpl,unbound} \end{aligned}$$

The calculations use artificial restraints, detailed below, to facilitate numerical convergence and connect the calculations with the standard concentration^16^, and the code accounts for the work of attaching and releasing ($\Delta G_{x,att}, \Delta G_{x,rel}, x = p, \, l$) these restraints as the system progresses from one state to another. BAT not only decouples the ligand from the bound and free states, but also turns off (“annihilates”) all intraligand electrostatic interactions during the decoupling process. This is because strong, unshielded, gas-phase, electrostatic attractions can cause serious conformational sampling problems, such as conformational locking by strong intramolecular hydrogen bonding.

The second alchemical method, simultaneous decoupling and recoupling (SDR), was previously introduced to compute the binding free energies of neutral and charged ligands to the glutamate receptor^27^. In SDR, $\Delta G_{trans}$ is computed as the reversible work of alchemically decoupling the ligand from the binding site and simultaneously recoupling it with the simulation system at a location in bulk solvent far from the protein. These calculations use the same set of restraints as the DD calculations. This approach prevents any change in the net charge of the simulation system, and thus avoids numerical artifacts associated with such charge changes^44^.

The BAT.py code also supports the attach-pull-release (APR) method, which computes the transfer free energy ${\Delta G_{trans}}$ through a physical pathway^23,24^. The APR method needs a low-barrier physical path along which the ligand can be pulled from the binding site to bulk solvent. This is straightforward for some ligands in surface binding-sites but is difficult to assure in all cases, especially for buried binding pockets. Nonetheless, the APR implementation can still be of value, so the Supporting Information (SI) describes the present implementation and provides an illustrative sample calculation.

## Methods

### Restraints and their corresponding free energies of attachment and release

The BAT code employs essentially the same set of restraints as previously described for the APR method^24^. Both the protein and the ligand are subject to two types of restraint. One type restrains the position and orientation of the molecule in the frame of reference of the simulation box. These translational and rotational (TR) restraints are constructed with added length, angle, and dihedral potential energy terms defined between atoms of the molecule (protein or ligand) and three dummy atoms, termed N1, N2 and N3, whose locations are fixed in the lab frame (Fig. 2). These keep the protein and ligand restrained relative to the simulation box, and thus to each other^24^. The other type of restraint is applied to internal degrees of freedom of the protein and ligand, so these limit conformational freedom. They are designed to reduce fluctuations during the calculation of the transfer free energy, and thus help convergence. This benefit must be weighed against the added computational cost of computing the attach and release free energies (Eq. 3). Note that the final free energy of binding should be the same, aside from numerical error, with or without the use of conformational restraints, and their use is, at least in principle, optional.

**Figure 4:**
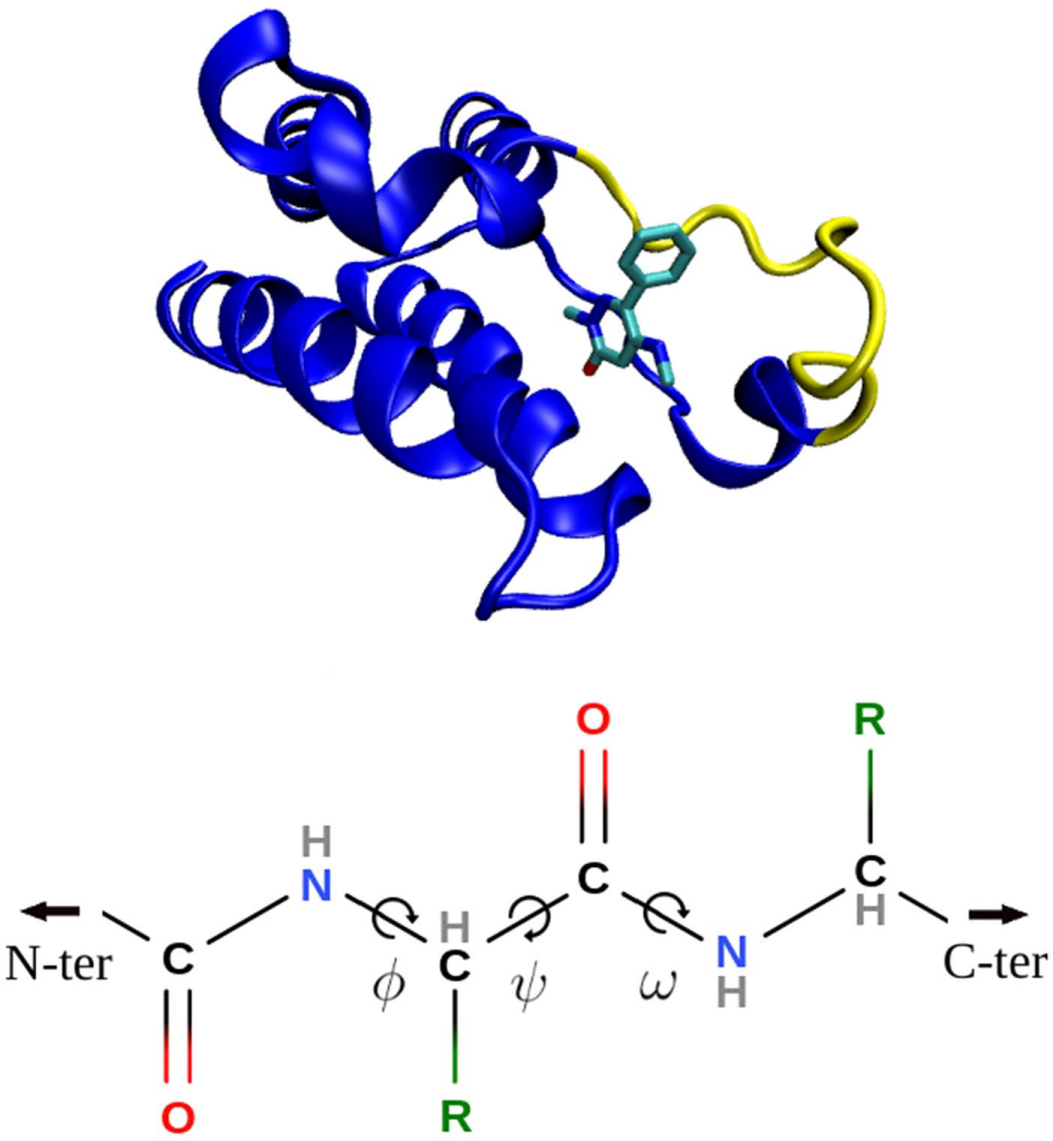
(Top) Second BRD4 bromodomain with the restrained backbone section (part of the ZA-loop) in yellow, the rest of the protein in blue, and the ligand colored by element. The structure is from the 5uf0 cocrystal structure. (Bottom) Protein backbone showing Ramachandran torsion angles. The optional backbone restraints in BAT.py are applied to only $\phi$ and $\psi$ angles.

#### Attachment and release of protein restraints

The TR restraints on the protein comprise harmonic potentials applied to one distance (D2), two angles (A3, A4), and three dihedrals (T4, T5, and T6), between the dummy atoms and three protein atoms (P1, P2, P3), which are termed the protein anchors^24^. These protein TR restraints are present and active during the equilibration phase and throughout most of the free energy calculation. They do not affect the protein’s conformational distribution, so there is no need to compute the free energy of attaching and releasing them. The force constant for the restraint on D2 is set in the BAT.py input file with the *rec_distance_force* variable, and the force constants for A3, A4, T4, T5, and T6, are set with the *rec_angle_force* parameter. The reference values of these restraints are taken from the starting conformation. (See user manual for details).

The conformational restraints on the protein comprise three harmonic distance restraints among the three protein anchors (P1, P2 and P3), to reduce TR fluctuations and coupling between TR motions and conformational changes. In addition, harmonic restraints may be applied to the backbone $\phi$ and $\psi$ angles in a user-selected range of protein residues (Fig.  4), in order to keep this section relatively rigid when transferring the ligand from binding site to bulk, particularly when using the APR method. Backbone $\omega$ angles are not restrained, as these are already rather rigid, due to the double-bond character of the peptide bond. This option is activated in the BAT.py input file using the *rec_bb* variable, with the chosen residue range defined by the *bb_start* and *bb_end* parameters. The spring constants for the protein distance and backbone dihedral conformational restraints are specified with the *rec_discf_force* and *rec_dihcf_force* variables, respectively.

The free energies of attaching, (${\Delta G_{p,att}}$), and releasing (${\Delta G_{p,rel}}$) the protein conformational restraints are calculated in the absence of the protein TR restraints (top and bottom processes in Fig.  2), using a number of simulation windows with $N_w$ values of the restraint spring constants, between zero and its full value, corresponding to $N_w$ windows. At each window, *i*, the spring constant for a given restraint, *r*, is defined by:

5 $$\begin{aligned} k_{ir} = \frac{attach\_rest(i)}{100}k_{fr} , \end{aligned}$$

where $k_{ir}$ is the spring constant, $k_{fr}$ is its full value defined in the input file, and *attach_rest(i)* is the multiplying factor associated with each window. These factors are defined by the *attach_rest* array in the BAT input file, and should go from 0 to 100. The same factors are used for all of the attach and release calculations for the protein and the ligand, which were determined and optimized in our previous APR study on the BRD4(1) protein^24^. Note, however, that the free energy of the ligand TR release is computed semi-analytically rather than with simulations. The free energies are obtained from the trajectories of all windows, using the multistate Bennett acceptance ratio (MBAR) method^48^, according to the equation:

6 $$\begin{aligned} G_{i}=-k_BT \sum \limits _{j=1}^{N_w} \sum \limits _{n=1}^{N_j} \frac{exp[-\beta U_{i}(\mathbf{r }_{n})]}{\sum \limits _{k=1}^{N_w} N_k exp[\beta G_{k} -\beta U_{k}(\mathbf{r }_{n})]} \end{aligned}$$

Here the subscripts *i*, *j*, and *k* index the simulation windows; *n* indexes the $N_j$ samples from window *j*, each with coordinates ${\mathbf{r }}_n$, and $N_k$ is, analogously, the number of samples from window *k*; $G_i$ and $G_k$ are the free energies of windows *i* and *k*, respectively; $\beta =1/k_{B}T$; $U_i(\mathbf{r }_{n})$ is the potential energy from the restraints defined in window *i* acting on the coordinates $\mathbf{r }_{n}$, which correspond to the *n*th sample from window *j*. Thus, $U_i(\mathbf{r }_{n})$ is given by

7 $$\begin{aligned} U_{i}(\mathbf{r }_{n}) = \sum \limits _{r=1}^R k_{ir}(x_{nr} - x_{0,ir})^2 , \end{aligned}$$

where *R* is the number of restraints being attached or released; $x_{nr}$ is the value in sample (or frame) *n* from window *j* of the internal coordinate (distance, angle, or torsion) corresponding to restraint *r*; and $k_{ir}$ and $x_{0,ir}$ are, respectively, the spring constant and equilibrium value for the *r*th harmonic restraint in window *i*. The program pyMBAR^48^ is invoked by BAT to solve Eq. (6) self-consistently for the free energies across the windows. The free energy difference between the initial (unrestrained) and final (fully restrained) states is then obtained directly from this set.

#### Attachment and release of ligand restraints

**Figure 5:**
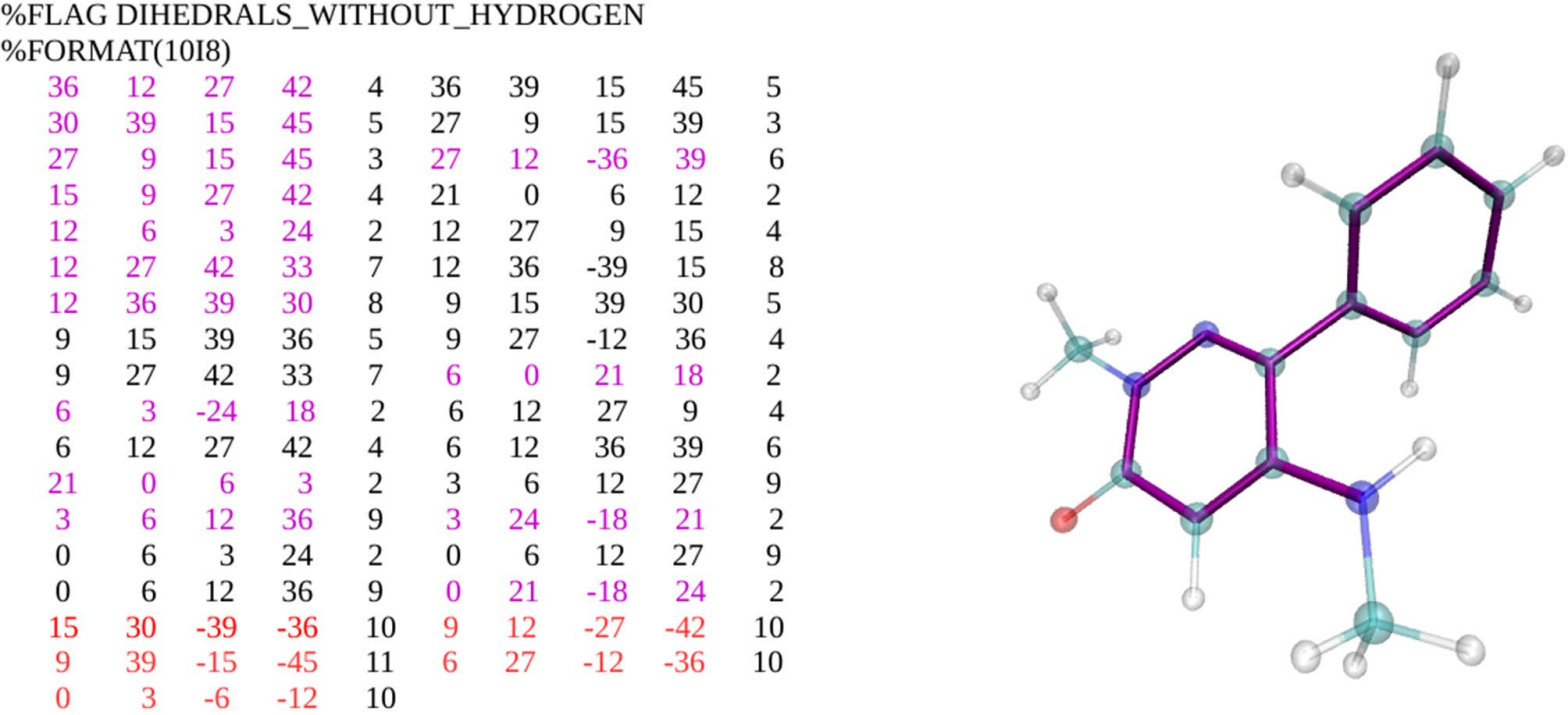
Example of ligand dihedral restraints, for PDB ID 5uf0. (Left) Section of AMBER parameter/topology file listing all the ligand dihedrals that do not include a hydrogen atom. Each row lists two torsions in terms of five indices; the first four map to specific atoms and the fifth maps to the associated force field parameters. Dihedrals restrained in the BAT procedure are highlighted in purple font, with redundant ones in black and improper dihedrals in red. (Right) Ligand from 5uf0 with restrained torsions highlighted with purple bonds. Cyan: carbon. White: hydrogen. Red: oxygen. Blue: nitrogen.

Like the protein, the ligand is subject to harmonic TR and conformational restraints^24^. The TR restraints again comprise a distance, D1, two angles, A1 and A2, and three torsions, T1, T2, T3, defined relative to the three fixed dummy atoms N1, N2 and N3. The spring constant for D1 is set in the BAT.py input file using the *lig_distance_force* variable. For the angle and dihedral TR restraints, the spring constant is defined by the *lig_angle_force* parameter. The reference values of these restraints are taken from the initial coordinates. The ligand conformational restraints include harmonic potentials on the three distances between its anchor atoms L1, L2 and L3 (Fig. 2). In addition, essentially all dihedral angles are also restrained to make each ligand rigid and thereby accelerate convergence. For simplicity, torsions within rings are not excepted from the set of restraints, although they are not always necessary. The BAT.py script automatically assigns these restraints for each ligand. It uses the ligand’s AMBER parameter/topology (prmtop) file (Fig. 5) to identify all proper dihedral terms not involving a hydrogen atom, and assigns a restraint to one arbitrarily chosen dihedral term for each central bond. The spring constants for the ligand’s internal distance and dihedral restraints are set in the BAT.py input file via the *lig_discf_force* and *lig_dihcf_force* parameters, respectively, and their reference values are taken from the starting coordinates. Fig. 5 illustrates the assignment of 14 conformational dihedral restraints for the ligand from cocrystal structure 5uf0.

The free energies of attaching and releasing the ligand restraints may be separated into conformational and TR parts:

8 $$\begin{aligned} \Delta G_{l,att}= & {} \Delta G_{l,conf,att} + \Delta G_{l,TR,att} \end{aligned}$$

9 $$\begin{aligned} \Delta G_{l,rel}= & {} \Delta G_{l,conf,rel} + \Delta G_{l,TR,rel} \end{aligned}$$

During the attachment stage (*att*), the ligand is in the binding site of the restrained protein. The conformational restraints are applied first, yielding the free energy change $\Delta G_{l,conf,att}$ for making the ligand essentially rigid. The TR restraints are then applied, yielding the free energy change ($\Delta G_{l,TR,att}$) for restraining the ligand in the binding site. During the release stage (*rel*), $\Delta G_{l,conf,rel}$ is computed with the ligand in a separate simulation box with no TR restraints present. The values of $\Delta G_{l,conf,att}$, $\Delta G_{l,TR,att}$, and $\Delta G_{l,conf,rel}$ are calculated the same way as the protein conformational restraints, using MBAR (Eqs. (5)–(7)), with simulation windows having intermediate values of the harmonic spring constants, also defined by the *attach_rest* input array. The final term in Eq. (9), $\Delta G_{l,TR,rel}$, is calculated by numerical quadrature of the following integral, which is based on Euler angles and spherical coordinates:

10 $$\begin{aligned} \Delta G_{l,TR,rel}= & {} k_{B}Tln \left( {\frac{C^\circ }{8 \pi ^2}} \right) + k_{B}Tln \int \limits _{0}^{\infty } \int \limits _{0}^{\pi } \int \limits _{0}^{2\pi } exp[-\beta (u_r + u_\theta + u_\phi )]r^{2}sin\theta d\theta d\phi dr \nonumber \\&\quad +k_{B}Tln \int \limits _{0}^{\pi } \int \limits _{0}^{2\pi } \int \limits _{0}^{2\pi } exp[-\beta (u_\Theta + u_\Phi + u_\Psi )]sin\Theta d\Theta d\Phi d\Psi \end{aligned}$$

Here $C^\circ$ is the standard concentration, 1 M = 1/1661Å$^3$, and *r*, $\theta$ and $\phi$ are the distance D1, angle A1, and dihedral T1, respectively^24^. In the last term on the right, which integrates over ligand orientation, $\Theta$ is the angle A2, $\Phi$ is the dihedral T2, and $\Psi$ is the dihedral T3. These are three Euler angles which define the orientation of the ligand in space. The harmonic potential applied to the distance *r* has the form:

11 $$\begin{aligned} u_r(r) = k_d(r - r_{0})^2 , \end{aligned}$$

with $k_d$ the *lig_distance_force* spring constant and $r_0$ its reference value. A similar expression is used for restrained angles and dihedrals:

12 $$\begin{aligned} u_a(r) = k_a(a - a_{0})^2 , \end{aligned}$$

with *a* a given angle/dihedral, $k_a$ the *lig_angle_force* spring constant, and $a_0$ its reference value. The value of $r_0$ is the reference distance, D1, from dummy atom N1 to ligand atom L1, in the bound state; this is always sets to 5.00 Å by construction (“Anchor atoms and dummy atoms”).

### Transfer of ligand from binding site to bulk solvent

The DD method uses two non-physical paths to calculate the transfer free energy of the fully restrained ligand from binding site to bulk, $\Delta G_{trans}$ (Eq. 4 and Fig. 3). Here, each of the terms on the right side of Eq. (4) is further separated into an electrostatic component and a Lennard–Jones component:

13 $$\begin{aligned} \Delta G_{dcpl,bound}= & {} \Delta G_{elec,bound} + \Delta G_{LJ,bound} , \end{aligned}$$

14 $$\begin{aligned} \Delta G_{dcpl,unbound}= & {} \Delta G_{elec,unbound} + \Delta G_{LJ,unbound} , \end{aligned}$$

Here $\Delta G_{elec,bound}$ is the free energy change for discharging all the atomic partial charges of the bound ligand, and ${\Delta G_{LJ,bound}}$ is the free energy change for turning off all LJ interactions between the (electrically discharged) bound ligand and its environment. This process turns off all intraligand electrical interactions, but preserves the intra-ligand LJ interactions. The LJ decoupling term is computed with soft-core potentials as implemented in AMBER, in order to smoothly switch off the LJ interactions and thus avoid numerical problems at the transformation end-points. The free energy for ligand decoupling in bulk, ${\Delta G_{dcpl,unbound}}$, follows the same decoupling procedures, but for the ligand in a simulation box of solvent without the protein. Running the decoupling calculations for the ligand with its conformational restraints present avoids numerical challenges that can otherwise result from large changes in the conformational preferences of the ligand between the coupled and decoupled (gas phase) states^49^. The BAT software allows these decoupling free energies to be computed via thermodynamic integration with Gaussian quadrature (TI-GQ)^43^ or MBAR, using the pyMBAR software^50^, based on outputs available from the AMBER simulations. The choice of method is dictated by the *dd_type* parameter in the BAT input file.

The SDR method involves decoupling the ligand from the binding site while simultaneously recoupling it to the bulk solvent in the same simulation box, so that the net charge of the system remains constant during the transformation. While one restrained ligand is decoupled in the binding site, another identical ligand restrained far from the protein is coupled back to the solvent, for both the electrostatic and the LJ components. That way the $\Delta G_{elec,bound}$ and $\Delta G_{elec,unbound}$ are calculated simultaneously in the same box, as are $\Delta G_{LJ,bound}$ and $\Delta G_{LJ,unbound}$, giving:

15 $$\begin{aligned} \Delta G_{elec}= & {} \Delta G_{elec,bound} - \Delta G_{elec,unbound} , \end{aligned}$$

16 $$\begin{aligned} \Delta G_{LJ}= & {} \Delta G_{LJ,bound} - \Delta G_{LJ,unbound} , \end{aligned}$$

When using this simultaneous approach, the extra variable *dd_dist* has to be added to the input file, which defines the *z* distance between the bound ligand and its bulk counterpart. Its value will depend on the size of the protein and the position of the binding site, and has to be chosen so that the ligand-protein interactions are negligible when the former is in bulk.

### Calculation definition and setup

As noted in the functionality and workflow section, BAT can accept as input either a single protein-ligand cocrystal structure or a single protein structure and a set of ligand poses generated with a docking program. As detailed in the user manual, the *calc_type* parameter is used to choose between these. The processing of the input structures to generating computed binding free enegies is detailed in the following subsections.

#### Anchor atoms and dummy atoms

The user identifies the desired protein anchor atoms (P1, P2 and P3) with the *P1*, *P2* and *P3* variables, and provides a reference protein structure for the orientation of the target protein. It is important that the protein anchor atoms be chosen to work for the reference structure, as any additional structures of the protein will be aligned to it in the source of the free energy calculations. The alignment of the complex relative to the protein reference structure is done with the program MUSTANG^46^, by first aligning the two protein sequences and then finding the optimal superposition of the two structures. Thus, the reference structure does not need to have the exact same sequence as the target one, so one may use only one reference for a set of similar proteins, with equivalent residues as the three protein anchors. The BAT.py procedure automatically assigns the ligand anchor atoms, L1, L2 and L3, and sets up the coordinates of the N1, N2 and N3 dummy atoms. This is done using a procedure that avoids possible gimbal-locking, which could result if the angle between three adjacent atoms of a given dihedral approached 0$^\circ$ or 180$^\circ$. Gimbal-locking can cause large restraint forces, leading to instabilities and crashes during the simulation, and therefore should be avoided.

**Figure 6:**
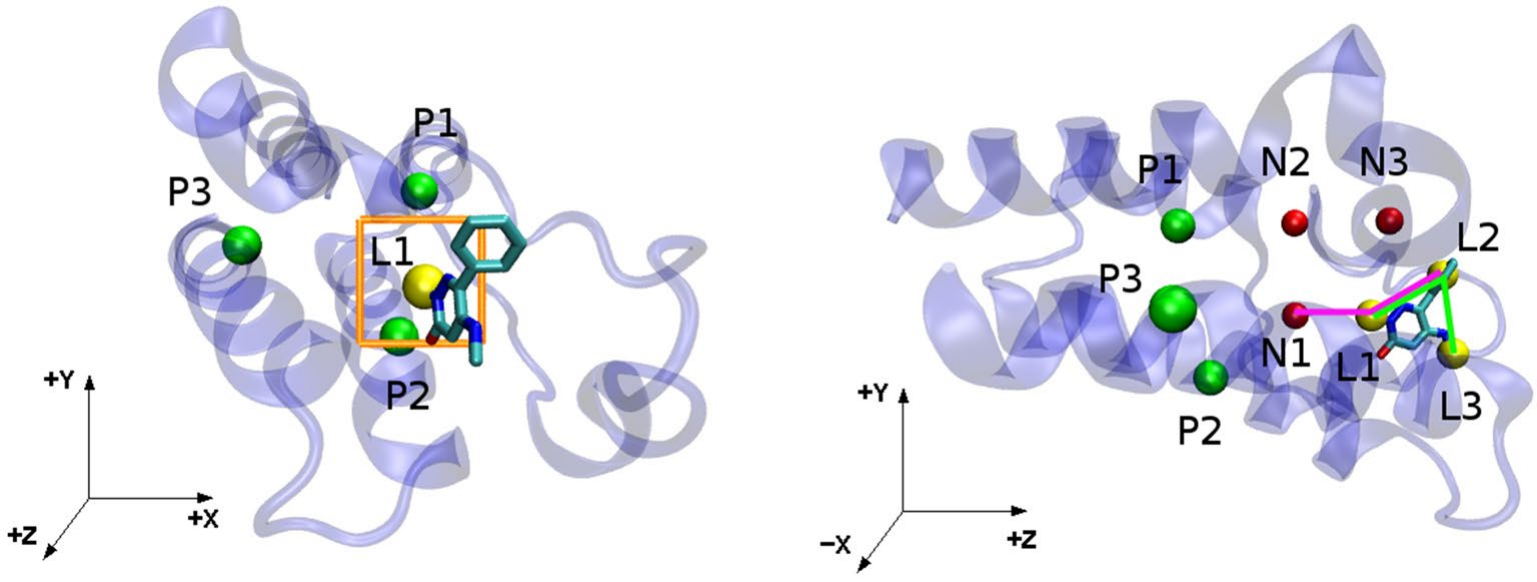
Definition of ligand anchor atoms and dummy atoms for the co-crystal structure 5uf0. (Left) Strike zone (orange square), ligand anchor atom L1 (yellow), and protein anchor atoms (green). (Right) Same system rotated relative to the left-hand panel, illustrating the definition of the L2 and L3 ligand anchor atoms (yellow). The N1–L1–L2 and L1–L2–L3 angles are shown with green and purple lines, respectively, and dummy atoms are shown in red.

The aligned protein-ligand complex is used to select the ligand anchor atoms and position the dummy atoms. First a “strike zone” is defined for use in identify the L1 ligand atom (Fig. 6, left). The strike zone is a square of side-length 2*l1_range, oriented perpendicular to the *z* axis. The center of this square has *x* and *y* coordinates given by $x_{P\textit{1}}+l\textit{1}\_{x}$ and $y_{P\textit{1}}+l\textit{1}\_{y}$, respectively, where $x_{P\textit{1}}$ and $y_{P\textit{1}}$ are the *x* and *y* coordinates of atom P1 (Fig. 6). Here *l1_x*, *l1_y* and *l1_range* are user-defined input parameters. Guidelines for selecting these parameters are provided in the user manual. The L1 anchor atom will be the ligand atom with *x* and *y* coordinates inside the strike zone and with the lowest *z* distance from P1, with the requirement that this distance is between the user-defined parameters *l1_z* (minimum) and *l1_zm* (maximum). The minimum value ensures that the N1 dummy atom can be placed between P1 and L1 in the *z* axis, and the maximum value avoids finding L1 if the ligand has left the binding site.

The first dummy atom, N1, is assigned the same *x* and *y* values as L1 but with a *z* distance of 5.0 Å from L1. The whole system is now rotated around the *z* axis, so that P1, N1 and L1 have the same *x* coordinates and thus are all located in the same *yz* plane. The second dummy atom, N2, is then placed with the same *z* value as N1, but with the *x* and *y* coordinates of P1. Dummy atom N3 is then positioned with the same *x* and *y* coordinates as N2, but with a distance from N2 in the *z* axis having the same value as the magnitude of the N1–N2 distance. Now P1, L1, N1, N2 and N3 are all located in the *yz* plane, as shown in the right hand panel of Fig. 6.

The remaining ligand anchor atoms, L2 and L3, are now selected. The L2 anchor is defined as the ligand atom which provides an N1–L1–L2 angle as close as possible to 90$^\circ$, while having an L1–L2 distance between the minimum and maximum specified in the input file as *min_adis* and *max_adis*, respectively. Setting a minimum distance between anchors L1 and L2 prevents the application of excessively large forces on dihedral restraints, which could result from a small lever arm. The maximum distance parameter is not as critical, but is meant to avoid choosing ligand anchor atoms far from the binding site. If no L2 atom can be found inside the specified distance range, as may occasionaly occur if the ligand is very small, the two parameters can be adjusted in the BAT.py input file. (If one finds this happens too often for a given application, the availability of the Python code affords a skilled user the opportunity to develop a custom version that, for example, automates the optimization of these parameters for each ligand.) The analogous protocol is then used to choose L3 based on the positions of L1 and L2, but now keeping the L1–L2–L3 angle as close as possible to 90$^\circ$ and the L2–L3 distance within the specified distance range. The input parameters listed here only have to be defined once for a new protein system, and generally will work for any ligand that binds to the same binding pocket. This protocol ensures that the N2–N1–L1 and N1–N2–P1 angles are 90$^\circ$, minimizing the forces applied by the T1 and T4 dihedral restraints on the P1 and L1 atoms^24^.

#### Force field options, solvation, and ionization

The protonation states of the protein’s ionizable groups are predetermined by the user-selected residue templates. Protonation states of the ligand are set with the program OpenBabel^45^, for a pH chosen using the *ligand_ph* variable. The current BAT software automates free energy calculations using any AMBER protein force field, chosen with the *receptor_ff* variable. Ligand parameters are assigned with the AM1-BCC charge model^51^ and either version 1 or 2 of the General AMBER Force Field (GAFF)^52,53^, chosen with the *ligand_ff* input variable. Currently supported options for the water model, chosen with the *water_model* parameter, are TIP3P^54^, TIP4PEw^55^ or SPC/E^56^. The types of any dissolved ions are specified with the *cation* and *anion* input variables, and the ions are assigned Joung and Cheatham parameters appropriate to the selected water model^57^. This selection could easily be changed by modification of the Python code.

The Ambertools tleap software^42,43^ is used to solvate the protein-ligand complex and the dummy atoms in a box of water molecules. The BAT.py code allows the user to choose the number of water molecules in the box and the water padding in the *x* and *y* axes, using the *num_waters*, *buffer_x* and *buffer_y* parameters, respectively. The dependent variable is the water padding in the *z* direction, which is calculated using an efficient iterative relaxed Newton-Raphson approach, based on the cross-sectional *xy* area of the box, the requested number of water molecules, and the average tleap atomic density for each water model. With the *neutralize_only* keyword, dissolved counterions are added if needed for charge neutralization. With the *num_cations* parameter, a number of additional cations are also added for a desired ionic strength, with the same number of anions added for neutrality.

The binding free energy calculations also require simulations of the free protein and the free ligand. These calculations are automatically set up as follows. The size of the ligand box is set in the input file using the *lig_buffer* parameter; this defines the water padding in all three Cartesian axes. Counterions are added to neutralize the ligand, as needed. The variable *num_cat_ligbox* sets a user-defined number of additional cations, and the number of anions again is the dependent variable. The variables to create the box with the *apo* protein are the same used previously for the protein-ligand system, such as the number of waters, water model, *x* and *y* buffering, and number of cations.

### Simulation procedures

#### Energy minimization and heating

Each system prepared above is energy minimized, with the protein restraints fully turned on. Molecular dynamics is then run as the system is heated, over 100 ps, from 10 K to the desired simulation temperature of 298.15 K, at constant volume, and using a Langevin thermostat^58^ with a collision frequency of 1.0 ps$^{-1}$. Then a series of brief (15 ps) simulations is run with the pressure held at 1 bar with the Monte Carlo barostat^59^. This procedure allows the volume to adjust while avoiding possible crashes caused by excessive shrinking of the initial box. Once this step is concluded, the system is ready for all subsequent runs, which are performed at constant temperature and pressure. The simulation temperature, thermostat collision frequency and barostat type can be chosen using the *temperature*, *gamma_ln* and *barostat* variables, respectively.

#### Equilibration and preparation

This stage prepares each initial protein-ligand complex for the free energy calculations detailed in the previous sections. The first step is to relax the initial ligand-protein complex so that it either declares itself as unstable, and thus not worth further analysis, or else settles into a nearby free energy minimum which becomes the starting structure for the binding free energy calculation. A sequence of molecular dynamics simulations is run with weaker and weaker ligand TR restraints, but without any ligand conformational restraints, until the final simulation is performed with the ligand free in the binding pocket. The number of simulations, the scaling of the ligand TR force constants at each simulation, and the number of steps for each simulation, are defined with the variables *release_eq*, *eq_steps1* and *eq_steps2*. During this process, the protein TR restraints and the distance restraints among the P1, P2 and P3 anchors are maintained. If the protein backbone dihedral restraints are in use, one may either maintain them or turn them off so the protein can fully adapt to the docked ligand. This choice is controlled by the the *bb_equil* variable.

Once this relaxation is complete, an attempt is made to set up a fresh set of ligand restraints, using the procedures in the ligand restraints section. If an L1 anchor can no longer be identified inside the strike zone and within the maximum allowed P1-L1 *z* distance, *l1_zm*, the ligand is considered to have left the binding site during equilibration. The initial pose is then considered unstable, and no free energy calculation is done. However, if an L1 anchor can be identified, then the dependent anchors and dummy atoms are reassigned, all restraints are given reference values corresponding to the final equilibrated structure, and the simulation box is rebuilt, re-minimized, and re-equilibrated with all restraints in place. The number of MD steps in this second “preparation” equilibration is specified with *prep_steps1*. The resulting system structure is used to initiate calculation of the binding free energy components on the ligand-protein complex. For the components that have a separate box for the ligand, a new box is built using the same reference coordinates for the ligand conformation.

### Free energy calculations

The calculated binding free energy is a sum of contributions, using Eqs. (3), (4), (8), (9), (10), (13), and (14). Each window of each free energy calculation is independently equilibrated, and then a production simulation is used to collect data. The number of equilibration and production MD steps for all windows of each component are set using the *[component]_steps1* and *[component]_steps2* input parameters, respectively, where *[component]* is the letter code for the free energy component (Table 1), *steps1* is the number of equilibration steps, and *steps2* is the number of production steps.

**Table 1:** Letter codes used in specifying simulation parameters to be applied in computing the free energy contributions to ${\Delta G^{\circ }_{bind}}$.

Description	Letter	System	Method	Term
Attachment of protein conformational restraints	**a**	Complex	MBAR	${\Delta G_{p,att}}$
Attachment of ligand conformational restraints	**l**	Complex	MBAR	${\Delta G_{l,conf,att}}$
Attachment of ligand TR restraints	**t**	Complex	MBAR	${\Delta G_{l,TR,att}}$
Ligand charge annihilation in site (DD)	**e**	Complex	MBAR/TI-GQ	${\Delta G_{elec,bound}}$
Decoupling of ligand LJ in site (DD)	**v**	Complex	MBAR/TI-GQ	${\Delta G_{LJ,bound}}$
Decoupling of ligand LJ in bulk (DD)	**w**	Ligand only	MBAR/TI-GQ	${\Delta G_{LJ,unbound}}$
Ligand charge annihilation in bulk (DD)	**f**	Ligand only	MBAR/TI-GQ	${\Delta G_{elec,unbound}}$
Release of ligand TR restraints	**b**	Ligand only	Analytical	${\Delta G_{l,TR,rel}}$
Release of ligand conformational restraints	**c**	Ligand only	MBAR	${\Delta G_{l,conf,rel}}$
Release of protein conformational restraints	**r**	Protein only	MBAR	${\Delta G_{p,rel}}$

The SDR method does not include the **f** and **w** components, with components **e** and **v** having an extra ligand in bulk solvent. See main text and Eqs. (3), (4), (8),  (9), (10), (13), and (14) for definitions of the terms. System: the molecular system simulated to compute each term. Method: the free energy method used to compute each term.

Following completion of the simulations, BAT.py computes each free energy component using the methods listed in Table 1; i.e., TI-GQ, MBAR, and/or analytical. This analysis uses options set in the previous stages, such as *components*, *dd_type*, *lambdas*, and *weights*. The trajectories for every window are also split into *blocks* blocks and the free energies are computed separately for each block. This feature is helpful to check for convergence, as large variations across blocks signals convergence problems during the calculations. We also report the standard deviation across blocks as a conservative estimate of the uncertainty in each free energy term:

17 $$\begin{aligned} \sigma = \sqrt{ \frac{1}{N_b} \sum _{n=1}^{N_b} (x_n - {\bar{x}})^2}, \end{aligned}$$

Here ${\bar{x}}$ is the free energy calculated using the whole trajectory, $x_n$ is the free energy calculated for each block, $N_b$ is the number of blocks, and $\sigma$ is the standard deviation. The uncertainties computed in this way for each free energy term are added in quadrature to obtain the reported uncertainty of $\Delta G^\circ _{bind}$.

### Protein-ligand test systems

**Figure 7:**
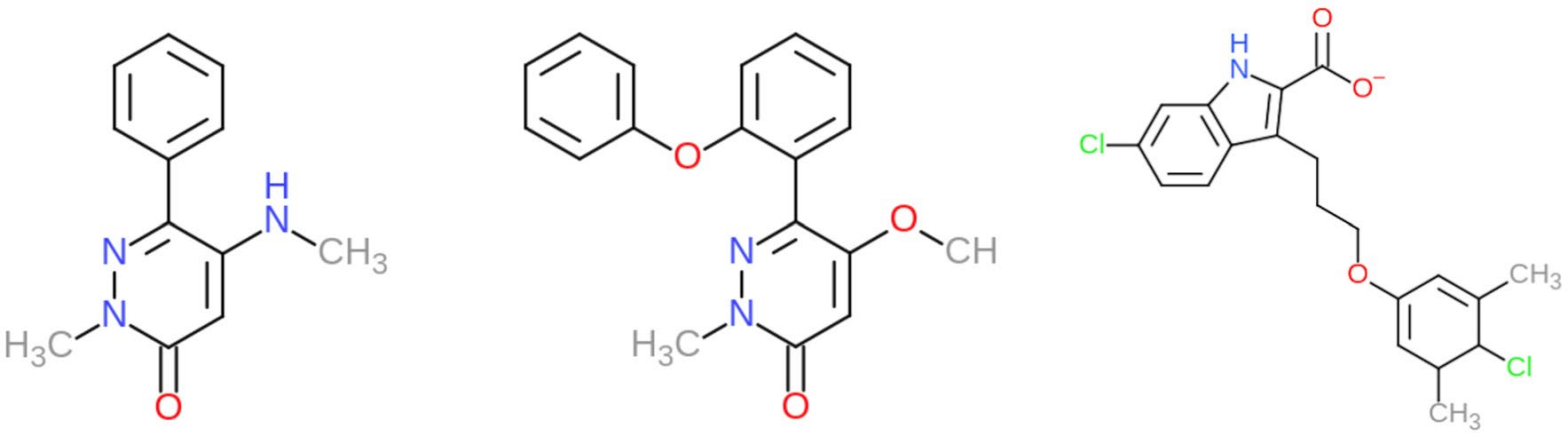
Chemical structures of ligands. (Left) BRD4 ligand 2-methyl-5-(methylamino)-6-phenylpyridazin-3(2H)-one (89J), for which binding free energies were computed. (Middle) Structure of the ligand, 5-methoxy-2-methyl-6-(2-phenoxyphenyl)pyridazin-3(2H)-one ligand, from the cocrystal structure 5uez, which provided the receptor structure for the 89J docking calculations. (Right) MCL-1 6-chloro-3-[3-(4-chloro-3,5- dimethylphenoxy) propyl]-1H-indole-2-carboxylic acid ligand (19H), evaluated in the second free energy calculations.

We tested DD free energy calculations with BAT.py for BRD4(2), the second bromodomain of the BRD4 protein, with the charge-neutral ligand 2-methyl-5-(methylamino)-6-phenylpyridazin-3(2H)-one (left of Fig. 7), which we will refer to as 89J, its PDB HETID. Binding free energies were computed for the cocrystal structure, 5uf0^60^, and also for five ligand poses generated by computational docking. This docking workflow uses a modified version of the one explained in the CELPPade tutorial^61,62^. Note that the docking workflow is not part of the BAT package and the docked poses it generated are included in the input examples associated with the present paper. The CELPPade tutorial shows how to participate in the challenge, by downloading the necessary data every week, running an example docking protocol using Autodock Vina, and uploading the predicted poses for each ligand once the docking is complete. Our modified docking script also uses Vina^63^ for the docking, as well as Chimera^64^ for protein and ligand setup and to convert the output files to pdb format. The protein structure used for docking is 5uez^60^, as this was identified by CELPP as having the ligand with the largest maximum common substructure (LMCSS) with our target ligand (middle of Fig. 7). The docked poses were characterized by calculating their structural RMSDs relative to the reference 5uf0 crystal structure, both before and after equilibration by MD. The program VMD was used to compute the RMSD values: the two protein structures were aligned and the RMSD Calculator plug-in was used, accounting for the symmetry of the phenyl group of the target ligand.

**Figure 8:**
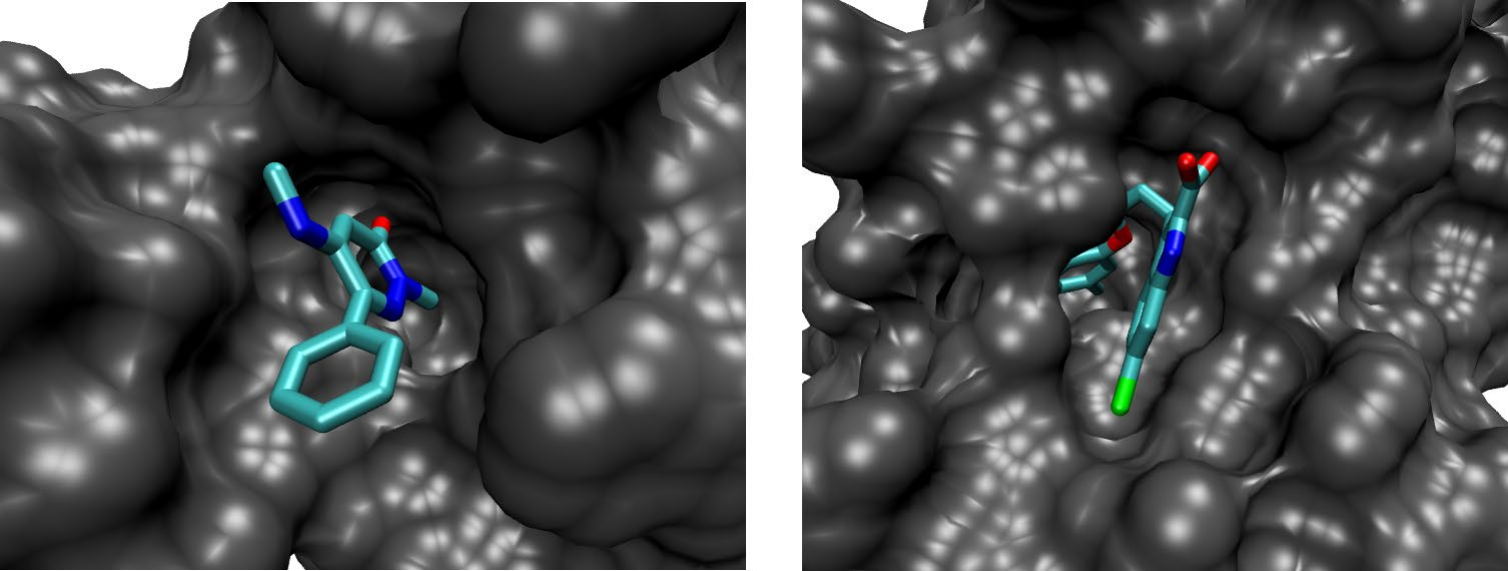
Crystal structures of proteins considered here. (Left) BRD4 crystal structure 5uf0, showing clear access of the ligand to the solvent. (Right) MCL-1 structure 4hw2, showing a site with possible steric barriers if using a physical path from the binding site to the solvent. The receptors are shown in gray, and the ligands are colored by element, with the chlorine atoms colored green.

We tested the SDR method for a different case, anionic ligand 6-chloro-3-[3-(4-chloro-3,5- dimethylphenoxy) propyl]-1H-indole-2-carboxylic acid molecule (PDB HETID 19H, right of Fig. 7)) binding to the human MCL-1 protein. The crystal structure of this complex, 4hw2^65^ , shows that the ligand does not have clear access out of the binding pocket (Fig. 8), so it would be difficult to study with the APR method. The SDR method was applied to the crystal pose as well as five poses generated by docking with the AutoDock Vina script included in the BAT.py distribution and explained in the User Guide. This does not need the CELPP workflow and can be performed on any receptor of choice. In order to present a realistic challenge, the docking was based on a structure solved with a different ligand, 6oqb^66^. The docked poses were evaluated as done for the 5uf0 case, above.

### Computational details

BAT.py is compatible with AMBER18 and AMBER20, which should be chosen using the *amber_version* variable, according to the *pmemd*.*cuda* version that will be employed for the simulations. The correct value for this variable is particularly important for the electrostatic decoupling processes, because in this case an additional set of ligand restraints has to be assigned if using AMBER20. In the present study, all simulations invoked by BAT.py use *pmemd*.*cuda* from AMBER18, with rectangular periodic simulation boxes. The AMBER “cut” parameter was set to 9.0 Å, and long-range electrostatics were calculated using the PME method. All bonds involving hydrogen were constrained by using the SHAKE algorithm^67^. The three dummy particles, N1, N2, and N3, are assigned zero charge, zero Lennard-Jones radius and well-depth, and a mass of 220 Da, with their Cartesian coordinates restrained by a harmonic force constant of $$k=50 kcal/(mol.$$Å$$^2)$$. We used hydrogen mass repartitioning (HMR)^68^ in all simulations, and an MD with a time step of 4 fs. In the HMR procedure, the hydrogen mass is multiplied by a factor of 3 and this enhanced hydrogen mass is subtracted from the atom to which the hydrogen is bonded. The use of HMR is optional, and is selected with the *hmr* variable. Other simulation options, such as the cutoff value, time step and output frequency, can be set in the BAT.py input file with the same variables used in the *pmemd*.*cuda* simulation input file. The input parameters for the binding free energy calculations, such as $$\lambda$$ values, simulation times, force constants and water model, are included in the Supporting Information (SI).

### Code availability

The calculations presented here are automated, given a set of basic input files and parameters, so they can be reproduced and generalized. The software is available on GitHub, as are a tutorial, a more complete User Guide, and the input files needed to replicate the present calculations^69^. Also included are the input parameters needed to run similar calculations on several other protein-ligand systems, and docking scripts that can be used to prepare the systems for BAT calculations. These scripts include the system preparation using Chimera, sample files, and a bash script to perform the docking automatically using AutoDock Vina.

## Results and discussion

We used BAT.py to carry out test calculations of the binding free energy of the two systems described in the previous sections. In this section we report on their accuracy and consider their potential to distinguish correct from incorrect poses, with the caveat that these two test cases can only serve as initial proof of principle. We then report the computational effort required for these calculations, and discuss the suitability of our software for use in a high-throughput scenario.

### Binding free energies and pose evaluation

#### Double decoupling calculations for BRD4

The binding free energy computed with the crystallographic pose is − 6.1 kcal/mol, a difference of − 0.9 kcal/mol when compared to the experimental result of − 5.2 kcal/mol^60^ (Table 2 and Table S1 from the SI). In addition, binding free energies computed with the two docked poses whose RMSDs are at most 2.0 Å (poses 2 and 5) show good consistency with the crystal structure result, and are within 1.5 kcal/mol of experiment (Table 2). However, binding free energies computed with the less accurate docked poses (1, 3, and 4), with initial RMSD values $\ge$ 5.4 Å), are more positive by at least 3 kcal/mol, and hence less favorable than those obtained with the more accurate poses (Table 2). Thus, the absolute binding free energy calculations yield reasonable agreement with experiment and distinguish accurate from inaccurate binding poses, as hoped. Interestingly, during the equilibration phase of the calculations, the RMSD of the crystallographic pose rose somewhat, from 0.0 to 1.3 Å, whereas both poses 2 and 5 moved closer to the crystallographic pose. Note, however, that these values are for single conformational snapshots, and that the RMSD values fluctuate in the course of a simulation.

The free energies reported in Table 2 used the TI-GQ method to compute the decoupling and annihilation terms, but we also ran these calculations with MBAR, and the two methods should give the same result in the limit of infinite sampling and infinitesimally spaced windows. Table 3 compares the results from these methods for each of the four decoupling or annihilation terms (Eqs. 13 and 14), for the crystal structure calculations represented in Table 2. The TI-GQ calculations used 12 windows, while MBAR used 23 windows between $\lambda =0$ and $\lambda =1$ (see SI). The number of steps for each window was the same in both methods, resulting in nearly twice as much simulation time for the MBAR calculations. The deviations between the two sets of calculations range from 0.1 to 0.4 kcal/mol for the four terms, showing good consistency between the two approaches. Note that the TI-GQ and MBAR calculations use independent sets of data, since the lambda values, and thus the simulation input parameters for each window, are not the same between the two. Table 3 also shows lower reported uncertainties for MBAR, when compared to TI-GQ. This could be due to the MBAR calculations having a greater number of lambda values between 0 and 1, and thus more sampling between the coupled and decoupled states.

#### Simultaneous decoupling and recoupling calculations for MCL-1

We first checked that the SDR method agrees with the DD method for BRD4 and its neutral ligand. As shown in Table 4 the differences between the electrostatic and LJ components of the transfer free energies from the two methods are within their respective numerical uncertainties. We then applied the SDR method to the MCL-1 system, with its charged ligand. As shown in Table 5 and Table S2 the binding free energy computed from the crystal structure is − 12.2 kcal/mol, which may be compared with the experimental value of − 10.0 kcal/mol^65^. Encouragingly, the two docked poses with RMSD < 2.0 Å relative to the crystallographic pose have similar computed binding free energies (− 12.5, − 12.0), while the three poses with larger RMSDs have computed binding free energies weaker than − 10 kcal/mol. Thus, the free energy calculations correctly identify the most accurate poses.

**Table 2:** Summary of computational results for the BRD4 (5uf0) system.

	Crystal	Pose 1	Pose 2	Pose 3	Pose 4	Pose 5
Initial RMSD	0.00	5.36	2.00	5.58	5.50	1.07
Equilibrated RMSD	1.30	5.26	0.45	5.33	4.23	0.74
$-{\Delta G^{\circ }_{bind}}$	6.1 (0.6)	2.5 (0.9)	6.7 (0.6)	2.6 (0.8)	1.5 (0.6)	6.5 (0.8)

Top two data rows give the structural root-mean-square deviations (RMSDs, Å) from the reference crystal pose in 5uf0, of the various ligand poses before (Initial) and after (Equilibrated) the MD equilibration step. The poses are numbered from the best-scoring (Pose 1) to worst-scoring (Pose 5) according to Vina. The last data row gives the computed binding free energies (kcal/mol) starting from each pose or the crystal structure. Uncertainties are provided in parentheses, according to Eq. (17). The tabulated double decoupling free energies were computed using TI with 12-point Gaussian quadrature; the other free energy terms were computed as shown in Table 1.

The present results illustrate the potential for physics-based, absolute binding free energy methods to distinguish accurate from inaccurate poses and to compute standard binding free energies that may be compared directly with experiment. Absolute binding free energy methods are particularly suitable for virtual screening, where the compounds of interest can be highly diverse. In contrast, relative binding free energy methods^9–13^, which involve alchemically changing one compound to another, are best suited to for comparing the affinities of chemically similar compounds, as in the scenario of lead optimization. In summary, although more testing is clearly needed, this initial study is encouraging and motivates future broader tests on other protein-ligand systems.

**Table 3:** Comparison of annihilation/decoupling free energies (kcal/mol) computed with MBAR and TI-GQ.

	MBAR	TI-GQ	Difference
${\Delta G_{elec,bound}}$	− 8.7 (0.3)	− 8.3 (0.5)	− 0.4
${\Delta G_{LJ,bound}}$	10.1 (0.2)	10.0 (0.2)	0.1
-${\Delta G_{LJ,unbound}}$	0.9 (0.1)	1.0 (0.1)	− 0.1
-${\Delta G_{elec,unbound}}$	11.1 (0.02)	11.0 (0.1)	0.1

Estimated uncertainties are given in parenthesis.

**Table 4:** Comparison of the electrostatic (${\Delta G_{elec}}$) and Lennard–Jones (${\Delta G_{LJ}}$) components of the transfer free energy ($\Delta G_{trans} = \Delta G_{elec} + \Delta G_{LJ}$) computed with the conventional DD method and the SDR method, both integrated with the TI-GQ approach.

	DD method	SDR method	Difference
${\Delta G_{elec}}$	2.7 (0.5)	3.4 (0.4)	− 0.7
${\Delta G_{LJ}}$	11.0 (0.3)	11.2 (0.2)	− 0.2

Estimated standard errors of the mean are given in parentheses.

**Table 5:** Summary of computational results for the MCL-1 (4hw2) system, using the simultaneous decoupling method.

	Crystal	Pose 1	Pose 2	Pose 3	Pose 4	Pose 5
Initial RMSD	0.00	6.50	6.97	1.87	1.46	4.52
Equilibrated RMSD	0.70	4.85	7.76	1.65	0.99	6.13
$-{\Delta G^{\circ }_{bind}}$	12.2 (0.8)	9.7 (0.9)	9.4 (0.5)	12.5 (0.7)	12.0 (1.0)	6.4 (1.2)

The columns and rows follow the same definitions as Table 2, also using TI with 12-point Gaussian quadrature for the decoupling free energies.

### Performance and costs

Detailed timings for the various components of the DD calculations on a single NVIDIA GTX 1070 GPU, for a single ligand pose, are provided in Table 6. Each calculation involved a total of 1.24 $\upmu$s of simulations, the same order of magnitude as reported for other recent ABFE calculations^19,20,70^. We anticipate that moving to the latest NVIDIA GPUs will roughly halve the wall-clock times reported here. It is also worth highlighting the potential for massive parallelization. Because each simulation window can be run independently, a trivial, full parallelization for one pose could reduce the wall-clock time by about two orders of magnitude, and further speedup can be achieved by trivial parallelization across poses. We estimate that combining new GPU technology with full parallelization will reduce the wall-clock time for a single ligand to about 3 h. It is also worth noting that the speed of a simulation with *pmemd*.*cuda* depends mainly on the choice of GPU, and far less on the choice of CPU, motherboard, etc. As a consequence, high-performance simulations can be achieved at low cost by using computers configured with strong GPUs but minimalist components otherwise.

**Table 6:** Computational speed and timings (wall clock) to run a binding free energy calculation with a single GTX 1070 GPU on a computer with no other calculations running.

Stage	Atoms	Speed	Simulation time (ST)	Computation time (CT)	ST per window	CT per window
**Free energy terms**						
Equilibration/ Preparation	$\sim$ 40k	$\sim$ 205 ns/d	80ns	0.39 d	–	–
${\Delta G_{p,att}}$	$\sim$ 40k	$\sim$ 205 ns/d	96ns	0.47 d	6ns	0.70 h
${\Delta G_{l,conf,att}}$	$\sim$ 40k	$\sim$ 205 ns/d	96ns	0.47 d	6ns	0.70 h
${\Delta G_{l,TR,att}}$	$\sim$ 40k	$\sim$ 205 ns/d	96ns	0.47 d	6ns	0.70 h
${\Delta G_{elec,bound}}$	$\sim$ 40k	$\sim$ 95 ns/d	28.8ns	0.30 d	2.4ns	0.61 h
${\Delta G_{LJ,bound}}$	$\sim$ 40k	$\sim$ 95 ns/d	288ns	3.03 d	24ns	6.10 h
${\Delta G_{LJ,unbound}}$	$\sim$ 6k	$\sim$ 490 ns/d	144ns	0.29 d	12ns	0.59 h
${\Delta G_{elec,unbound}}$	$\sim$ 6k	$\sim$ 450 ns/d	28.8ns	0.06 d	2.4ns	0.13 h
${\Delta G_{l,conf,rel}}$	$\sim$ 6k	$\sim$ 860 ns/d	192ns	0.22 d	12ns	0.33 h
${\Delta G_{p,rel}}$	$\sim$ 40k	$\sim$ 205 ns/d	192ns	0.94 d	12ns	1.40 h
**Total time**						
${\Delta G^{\circ }_{bind}}$	-	-	1.24 $\upmu$s	6.64 d	–	–

## Ready for high-throughput?

Our main goal in creating the BAT.py software is to enable rigorous ABFE calculations in a high-throughput regime and thus to enable their use in the first stages of drug discovery. To make that a reality, we believe two things are indispensable: automation of the free energy calculations and high performance of the simulations.

The first requirement is made possible by the workflow of BAT.py (Fig. 1), which can start either from a set of docked poses of a given ligand or from a crystal structure. Once the necessary input parameters are determined and optimized for a new receptor, along with a docking procedure to generate plausible poses, the calculations can be performed for a library of ligands by simply running BAT.py in the command line for each compound. The flexibility of BAT.py allows one to choose the optimal force-field parameters, adjust the simulation times to prioritize speed or exhaustive sampling, and set the most suitable protein conformational restraints. The second requirement is that the simulations can be performed with high performance and at low cost. This capability is now in view, given the fast *pmemd*.*cuda* implementation of AMBER and GPU implementations of other simulation packages, coupled with the increasing performance and affordability of GPUs.

Thus, these two conditions are now largely satisfied. This makes it possible to move to expanded testing of ABFE calculations as a tool for scoring docked ligand poses, while also estimating overall binding free energies, so that many ligands can be ranked in terms of their calculated affinities. We plan next to extend these calculations to other protein systems, testing different force fields, water models and simulation parameters, including in the context of the rolling CELPP pose-prediction exercise^62^. In future work, it may be useful to seek accelerated convergence through enhanced sampling techniques^71^ and hybrid Monte Carlo/MD methods that enable water to exchange between buried sites and the bulk solvent^72,73^.

## Supplementary information

Supplementary Information.
